# An Android for Emotional Interaction: Spatiotemporal Validation of Its Facial Expressions

**DOI:** 10.3389/fpsyg.2021.800657

**Published:** 2022-02-04

**Authors:** Wataru Sato, Shushi Namba, Dongsheng Yang, Shin’ya Nishida, Carlos Ishi, Takashi Minato

**Affiliations:** ^1^Psychological Process Research Team, Guardian Robot Project, RIKEN, Kyoto, Japan; ^2^Field Science Education and Research Center, Kyoto University, Kyoto, Japan; ^3^Graduate School of Informatics, Kyoto University, Kyoto, Japan; ^4^NTT Communication Science Laboratories, Nippon Telegraph and Telephone Corporation, Atsugi, Japan; ^5^Interactive Robot Research Team, Guardian Robot Project, RIKEN, Kyoto, Japan

**Keywords:** android, emotional facial expression, dynamic facial expression, Facial Action Coding System, robot

## Abstract

Android robots capable of emotional interactions with humans have considerable potential for application to research. While several studies developed androids that can exhibit human-like emotional facial expressions, few have empirically validated androids’ facial expressions. To investigate this issue, we developed an android head called Nikola based on human psychology and conducted three studies to test the validity of its facial expressions. In Study 1, Nikola produced single facial actions, which were evaluated in accordance with the Facial Action Coding System. The results showed that 17 action units were appropriately produced. In Study 2, Nikola produced the prototypical facial expressions for six basic emotions (anger, disgust, fear, happiness, sadness, and surprise), and naïve participants labeled photographs of the expressions. The recognition accuracy of all emotions was higher than chance level. In Study 3, Nikola produced dynamic facial expressions for six basic emotions at four different speeds, and naïve participants evaluated the naturalness of the speed of each expression. The effect of speed differed across emotions, as in previous studies of human expressions. These data validate the spatial and temporal patterns of Nikola’s emotional facial expressions, and suggest that it may be useful for future psychological studies and real-life applications.

## Introduction

Emotional interactions with other people are important for wellbeing (Keltner and Kring, 1998) but difficult to investigate in controlled laboratory experiments. While numerous psychological studies have presented pre-recorded photographs or videos of emotional expressions to participants and reported interesting findings regarding the psychological processes underlying emotional interactions (e.g., Dimberg, 1982), this method may lack the liveliness of real interactions, thus reducing ecological validity (Shamay-Tsoory and Mendelsohn, 2019; Hsu et al., 2020). Other studies used confederates as interaction partners and tested live emotional interactions (e.g., Vaughan and Lanzetta, 1980), but this strategy can lack rigorous control of confederates’ behaviors (Bavelas and Healing, 2013; Kuhlen and Brennan, 2013). Androids—that is, humanoid robots that exhibit appearances and behaviors that closely resemble those of humans (Ishiguro and Nishio, 2007)—could become an important tool for testing live face-to-face emotional interactions with rigorous control.

To implement emotional interaction in androids, the androids’ facial expressions must be carefully developed. Psychological studies have verified that facial expressions play a key role in transmitting information about emotional states in humans (Mehrabian, 1971). Studies of facial expressions developed methods for objectively evaluating facial actions (for a review, see Ekman, 1982), and the Facial Action Coding System (FACS; Ekman and Friesen, 1978; Ekman et al., 2002) is among the most refined of these methods. Based on observations of thousands of facial expressions in natural settings, together with a series of controlled psychological experiments, researchers identified the sets of facial action units (AUs) in the FACS corresponding to prototypical expressions of six basic emotions (Ekman and Friesen, 1975; Friesen and Ekman, 1983). For example, happy expressions involve an AU set consisting of the cheek raiser (AU 6) and lip corner puller (AU 12); surprised expressions involve the inner and outer brow raisers (AUs 1 and 2, respectively), the upper lid raiser (AU 5), and the jaw drop (AU 25). Numerous studies testing the recognition of photographs of facial expressions created based on this system verified that the expressions were recognized as the target emotional expressions above chance level across various cultures (e.g., Ekman and Friesen, 1971; for a review, see Ekman, 1993). Furthermore, the researchers described how the temporal aspects of dynamic emotional facial expressions are informative (Ekman and Friesen, 1975), which was supported by several subsequent experimental studies (for reviews, see Krumhuber et al., 2016; Dobs et al., 2018; Sato et al., 2019a). For example, Sato and Yoshikawa (2004) tested the naturalness ratings of dynamic changes in facial expressions and found that expressions that changed too slowly were generally rated as unnatural. Additionally, the effects of changing speeds differed across emotions, where fast and slow changes were regarded as relatively natural for surprised and sad expressions, respectively. Collectively, these psychological findings specify the spatial and temporal patterns of facial actions associated with facial expressions of emotions. Based on such findings, researchers have developed and validated novel research tools, including emotional facial expressions of virtual agents (Roesch et al., 2011; Krumhuber et al., 2012; Ochs et al., 2015). Virtual agents are promising tools to investigate emotional interactions with high ecological validity and control (Parsons, 2015; Pan and Hamilton, 2018). Androids may be comparably useful in this respect, and also have the unique advantage of being physically present (Li, 2015). If androids’ facial expressions can be developed and validated based on psychological evidence, they will constitute an important research tool for investigating emotional interactions.

However, although numerous studies have developed androids for emotional interactions (Kobayashi and Hara, 1993; Kobayashi et al., 2000; Minato et al., 2004, 2006, 2007; Weiguo et al., 2004; Ishihara et al., 2005; Matsui et al., 2005; Berns and Hirth, 2006; Blow et al., 2006; Hashimoto et al., 2006, 2008; Oh et al., 2006; Sakamoto et al., 2007; Lee et al., 2008; Takeno et al., 2008; Allison et al., 2009; Lin et al., 2009, 2016; Kaneko et al., 2010; Becker-Asano and Ishiguro, 2011; Ahn et al., 2012; Mazzei et al., 2012; Tadesse and Priya, 2012; Cheng et al., 2013; Habib et al., 2014; Yu et al., 2014; Asheber et al., 2016; Glas et al., 2016; Marcos et al., 2016; Faraj et al., 2021; Nakata et al., 2021; Table 1), few have empirically validated the androids that were developed. First, no study validated androids’ AUs coded using FACS (Ekman and Friesen, 1978; Ekman et al., 2002). Second, no study sufficiently demonstrated recognition of the six basic emotions conveyed by androids’ facial expressions. Many androids’ facial expressions were reportedly insufficiently developed to exhibit all six basic emotions (e.g., Minato et al., 2004). While several studies developed androids capable of exhibiting the six basic emotions, and recruited naïve participants to label the facial expressions, most did not statistically evaluate the accuracy (e.g., Kobayashi and Hara, 1993). One study conducted a statistical analysis that did not reveal significantly high level of recognition of disgust and fear (Berns and Hirth, 2006). Another study testing five basic emotions failed to observe better-than-chance recognition of fear (Becker-Asano and Ishiguro, 2011). Finally, no study systematically validated whether androids can show dynamic changes in facial expressions like humans. Only a few studies reported that incorporating the dynamic patterns of human facial expressions into an androids’ facial expressions led to high naturalness ratings of facial expressions during laughter (Ishi et al., 2019) and vocalized surprise (Ishi et al., 2017).

**TABLE 1:** Summary of studies on androids’ emotional facial expressions.

Study	Robot name	Emotional expression	Head DOF	Validation
Kobayashi and Hara, 1993	Face robot	6 basic emotions	24	Emotion recognition (no statistical test)
Kobayashi et al., 2000	–	Some (not specified)	21	–
Minato et al., 2004	Repliee R1	–	9	–
Weiguo et al., 2004	F&H robot	4 basic emotions	12	–
Ishihara et al., 2005	Affetto	Some (not specified)	12	–
Matsui et al., 2005	Repliee Q2	Some (not specified)	16	–
Berns and Hirth, 2006	ROMAN	6 basic emotions	21	Emotion recognition
Blow et al., 2006	KASPAR	Some (not specified)	8	–
Hashimoto et al., 2006	Saya	6 basic emotions	23	Emotion recognition (no statistical test)
Minato et al., 2006	CB^2^	Some (not specified)	14	–
Oh et al., 2006	Albert HUBO	Full range (not specified)	31	–
Sakamoto et al., 2007	Geminoid HI-1	–	13	–
Hashimoto et al., 2008	–	6 basic emotions	39	–
Lee et al., 2008	EveR-2	6 basic emotions	22	–
Takeno et al., 2008	Kansei	6 basic emotions	19	–
Allison et al., 2009	Brian	6 basic emotions	11	Emotion recognition (no statistical test)
Lin et al., 2009	Janet; Thomas	Various (not specified)	23	–
Kaneko et al., 2010	HRP-4C	Some (not specified)	11	–
Becker-Asano and Ishiguro, 2011	Geminoid F	5 basic emotions	12	Emotion recognition
Ahn et al., 2012	EveR-4 H33	13 basic emotions	33	Emotion recognition (no statistical test)
Mazzei et al., 2012	FACE	6 basic emotions	32	Emotion recognition (no statistical test)
Tadesse and Priya, 2012	–	Various (not specified)	–	–
Cheng et al., 2013	EVA	4 basic emotions	–	Emotion recognition (no statistical test)
Habib et al., 2014	PKD	Various (not specified)	24	–
Yu et al., 2014	–	6 basic emotions	13	Motion similarity; emotion recognition (no statistical test)
Lin et al., 2016	–	6 basic emotions	4	Emotion recognition (no statistical test)
Marcos et al., 2016	–	6 basic emotions	22	Emotion recognition (no statistical test)
Glas et al., 2016	ERICA	Wide range (not specified)	13	–
Asheber et al., 2016	–	6 basic emotions	8	–
Faraj et al., 2021	Eva	6 basic emotions	25	–
Nakata et al., 2021	Ibuki	7 basic emotions	18	–
This study	Nikola	6 basic emotions	35	FACS; emotion recognition; speed rating

*We included only androids that were human-like in appearance, and for which data were reported at conferences or in papers. DOF = degree of freedom; FACS = Facial Action Coding System.*

To resolve the issues described above, we developed an android head, called Nikola, and validated its facial actions and emotional expressions. Nikola has 35 actuators, designed to implement AUs relevant to prototypical facial expressions based on psychological evidence (Ekman and Friesen, 1975, 1978; Friesen and Ekman, 1983; Ekman et al., 2002). The temporal patterns of the actions can be programmed at a resolution of milliseconds. We conducted a series of psychological studies to validate Nikola’s emotional facial expressions. In Study 1, we applied FACS coding to Nikola’s single AUs, which underlie appropriate emotional facial expressions. In Study 2, we evaluated emotional recognition accuracy based on the spatial patterns of Nikola’s emotional facial expressions through an emotion labeling task. In Study 3, we evaluated the temporal patterns of Nikola’s dynamic facial expressions through a naturalness rating task.

## Study 1

Here, we used FACS coding for Nikola’s single facial actions. We expected that AUs specifically associated with the facial expressions corresponding to the six basic emotions to be produced.

### Materials and Methods

#### Development of the Android

Nikola was developed for the purpose of studying emotional interaction with humans. Currently, only the head and neck are complete; the body parts are under construction. It is human-like in appearance, similar to a male human child; it resembles a child to promote natural interactions with both adults and children. It is about 28.5 cm high and weighs about 4.6 kg. It has 35 actuators: 29 for facial muscle actions, 3 for head movement (roll, pitch, and yaw rotation), and 3 for eyeball control (pan movements of the individual eyeballs and tilt movements of both eyeballs). The facial and head movements are driven by pneumatic (air) actuators, which create safe, silent, and human-like motions (Ishiguro and Nishio, 2007; Minato et al., 2007). The pneumatic actuators are controlled by an air pressure control valve. The entire surface, except for the back of the head, is covered in a soft silicone skin. Video cameras are mounted inside the left and right eyeballs. Nikola is not a stand-alone system; the control valves, air compressor, and computer for controlling the actuators and sensor information processing are external.

The facial muscle actuators’ locations were selected to produce as many AUs as possible, specifically those associated with emotional facial expressions (Ekman and Friesen, 1975, 1978; Friesen and Ekman, 1983; Ekman et al., 2002), together with the information provided by previously constructed androids (Minato et al., 2004, 2006, 2007; Matsui et al., 2005; Glas et al., 2016). Specifically, we designed Nikola to produce the following AUs corresponding to the emotional expressions associated with six basic emotions: 1 (inner brow raiser), 2 (outer brow raiser), 4 (brow lowerer), 5 (upper lid raiser), 6 (cheek raiser), 7 (lid tightener), 10 (upper lip raiser), 12 (lip corner puller), 15 (lip corner depressor), 20 (lip stretcher), 25 (lips part), and 26 (jaw drop). Although AUs 9 (nose wrinkler), 17 (chin raiser), and 23 (lip tightener) are reportedly relevant to prototypical facial expressions (Ekman and Friesen, 1975; Friesen and Ekman, 1983), these AUs were not implemented owing to the technical limitations of the silicone skin. AUs 14 (dimpler), 16 (lower lip depressor), 18 (lip pucker), 22 (lip funneler), and 43 (eyes closed) were also designed to implement other communication-related facial actions (e.g., speech and blinking).

#### Procedure

We programmed Nikola to exhibit AUs on an individual basis. A certified FACS coder scored the AUs from the neutral status to the action apex using FACS (Ekman et al., 2002). When the AU was detected, the coder evaluated it according to five discrete levels of intensity (A: trace, B: slight, C: marked/pronounced, D: severe, and E: extreme/maximum) according to FACS guidelines (Ekman et al., 2002). The coder could view the sequence repeatedly by adjusting the program settings. The Supplementary Material provides video clips of these AUs.

### Results

**FIGURE 1:**
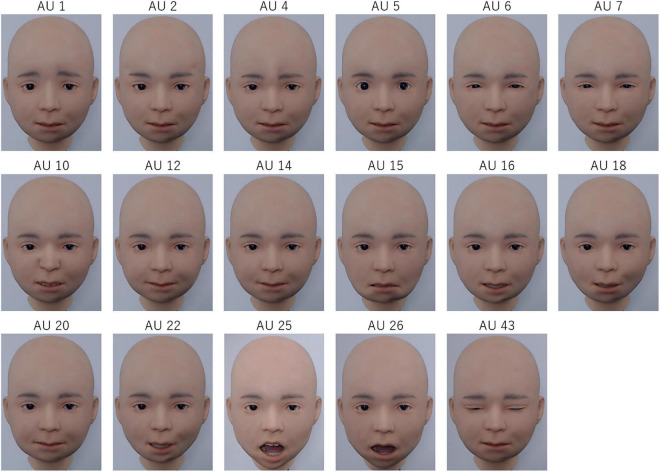
Illustrations of the facial action units (AUs) produced by the android Nikola. For AU 25, AU 25 + 26 is shown.

The AUs produced by Nikola are illustrated in Figure 1, and the results of the FACS coding are presented in Table 2. Figure 1 demonstrates that Nikola is capable of performing each AU. It was difficult to distinguish between AUs 6 (cheek raiser) and 7 (lid tightener), but the eyes’ outer corners were slightly lowered in AU 6. The maximum intensity of the AUs ranged from A (e.g., AU 12) to E (e.g., AU 26).

**TABLE 2:** Results of the Facial Action Coding System (FACS) coding of Nikola’s facial actions.

AU	AU description	Maximum intensity
1	Inner brow raiser	C
2	Outer brow raiser	B
4	Brow lowerer	C
5	Upper lid raiser	C
6	Cheek raiser	B
7	Lid tightener	D
10	Upper lip raiser	D
12	Lip corner puller	A
14	Dimpler	B
15	Lip corner depressor	B
16	Lower lip depressor	B
18	Lip pucker	A
20	Lip stretcher	B
22	Lip funneler	A
25	Lips part	E
26	Jaw drop	E
43	Eyes closed	E

*AU = action unit.*

### Discussion

Our results demonstrated that Nikola was capable of producing each AU based on manual FACS coding performed by a certified FACS coder. The results are consistent with several earlier studies’ findings that androids could exhibit AUs designed based on FACS (e.g., Kobayashi and Hara, 1993), but none of these studies involved evaluation by certified FACS coders. The coder found it difficult to differentiate AUs 6 (cheek raiser) and 7 (lid tightener). This is in line with earlier findings that androids struggled to replicate *z*-vector movements, including wrinkles and tension, compared with human expressions (Ishihara et al., 2021), owing to the physical constraints of artificial skin materials. The results of our intensity evaluation revealed that some AUs’ maximum intensities were not realized. This resulted from technical limitations, such as an insufficient number of actuators and skin materials. Collectively, the data suggest that Nikola can produce AUs associated with prototypical facial expressions, albeit with limited intensity.

## Study 2

Next, we devised prototypical facial expressions for Nikola reflecting six basic emotions and asked naïve participants to label photographs of these expressions, as in earlier psychological studies using photographs of human facial expressions as stimuli (Sato et al., 2002, 2009; Kubota et al., 2003; Uono et al., 2011; Okada et al., 2015). Because earlier studies of human expression stimuli consistently demonstrated emotion recognition above the level of chance, as well as differences across emotions (such as lower recognition rates for angry, disgusted, and fearful expressions than happy, sad, and surprised expressions), we expected such patterns to be seen with respect to emotion recognition of Nikola’s facial expressions.

### Materials and Methods

#### Participants

Thirty adult Japanese participants participated in this study (18 females; mean ± *SD* age, 36.0 ± 7.2 years). The sample size was determined based on an *a priori* power analysis using G*Power software ver. 3.1.9.2 (Faul et al., 2007). Assuming an α level of 0.008 (i.e., 0.05 Bonferroni-corrected for six tests), a power of 0.80, and a strong effect size (*d* = 0.8) based on an earlier study (Sato et al., 2002), the results indicated that 23 participants were required for a one-sample *t*-test. Participants were recruited through web advertisements distributed *via* CrowdWorks (Tokyo, Japan). After the procedures had been explained, all participants provided written informed consent to participate in the study, which was approved by the Ethics Committee of RIKEN. The experiment was performed in accordance with the Declaration of Helsinki.

#### Stimuli

**FIGURE 2:**
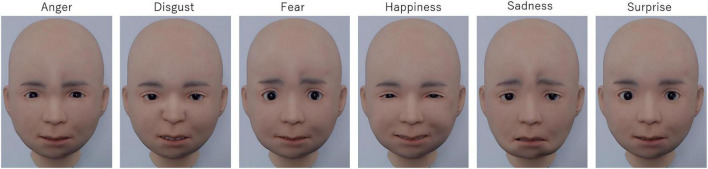
Illustrations of the facial expressions of six basic emotions produced by the android Nikola.

Six photographs of facial expressions depicting the six basic emotions (anger, disgust, fear, happiness, sadness, and surprise) produced by Nikola were used as stimuli (Figure 2). The facial expressions were produced by activating the AUs according to the Emotional Facial Action Coding System (EMFACS; Friesen and Ekman, 1983). The activated AUs included 4, 5, 7, and 23 for anger; 15 for disgust; 1, 2, 4, 5, 7, 20, and 26 for fear; 6 and 12 for happiness; 1, 4, and 15 for sadness; and 1, 2, 5, and 26 for surprise. The facial expressions of the six basic emotions were photographed using a digital web camera (HD1080P; Logicool, Tokyo, Japan). The photographs were cropped to 630 horizontal × 720 vertical pixels.

#### Procedure

The experiment was conducted *via* the Qualtrics online platform (Seattle, WA, United States). A label-matching paradigm was used, as in an earlier study (Sato et al., 2002). The photographs of Nikola’s facial expressions of the six basic emotions were presented on the monitor individually, and verbal labels for the six basic emotions were presented below each photograph. Participants were asked to select the label that best described the emotion shown in each photograph. No time limits were set, and no feedback on performance was provided. An image of each emotional expression was presented twice, pseudo-randomly, resulting in a total of 12 trials for each participant. Prior to the experiment, the participants performed two practice trials.

#### Data Analysis

The data were analyzed using JASP 0.14.1 software (JASP Team, 2020). Accuracy percentages for emotion recognition were tested for the difference from chance (i.e., 16.7%) using one-sample *t*-tests (two-tailed) with the Bonferroni correction; the alpha level was divided by the number of tests performed (i.e., 6). The emotion recognition accuracy data were also subjected to repeated-measures analysis of variance (ANOVA) with emotion as a factor to test for differences among emotions. The assumption of sphericity was confirmed using Mauchly’s sphericity test (*p* > 0.10). Multiple comparisons were performed using Ryan’s method. All results were considered statistically significant at *p* < 0.05.

### Results

**FIGURE 3:**
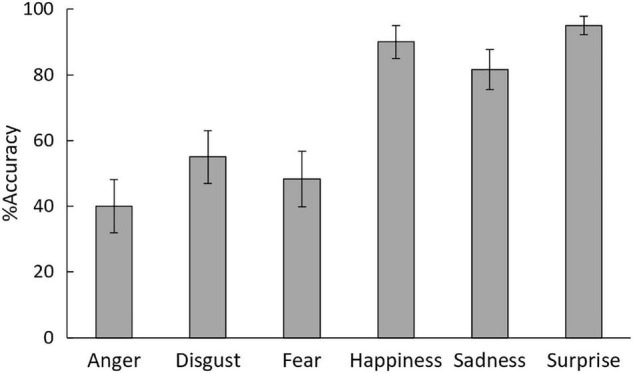
Mean (±*SE*) accuracy percentages for the recognition of six emotions in facial expressions in Study 2.

One-sample *t*-tests revealed that the accuracy percentage of emotionapl expression recognition for all emotion categories (Figure 3) was greater than chance, *t*(29) = 2.88, 4.74, 3.74, 14.58, 10.64, and 28.11; Bonferroni-corrected *p* = 0.042, 0.000, 0.007, 0.000, 0.000, and 0.000; Cohen’s *d* = 0.90, 1.24, 1.04, 3.27, 2.44, and 6.23 for anger, disgust, fear, happiness, sadness, and surprise, respectively.

The ANOVA with emotion as a factor for recognition accuracy revealed a significant main effect of emotion, *F*_(5, 145)_ = 15.94, *p* = 0.000, η^2^_*p*_ = 0.36. Multiple comparisons indicated that surprised, sad, and happy expressions were recognized with greater accuracy than disgusted, fearful, and angry expressions, *t*(245) > 3.21, *p* < 0.005.

### Discussion

Our findings indicated that the emotion recognition accuracy of Nikola’s facial expressions for all six basic emotions was above chance level. These results are consistent with earlier studies reporting that participants could recognize emotions from the facial expressions of androids, although the studies either did not determine whether recognition accuracy was better than chance (e.g., Kobayashi and Hara, 1993) or failed to find significantly higher recognition than chance for some emotions (Berns and Hirth, 2006; Becker-Asano and Ishiguro, 2011). Additionally, the results revealed differences in the accuracy of emotional recognition across emotional categories, with better recognition seen for happy, sad, and surprised expressions than for angry, disgusted, and fearful expressions. The results are consistent with earlier studies on emotion recognition using human facial expression stimuli (e.g., Uono et al., 2011). Compared with earlier studies using human stimuli, the overall emotion recognition percentage using photographs of Nikola as stimuli was low [e.g., 98.2 and 90.0% recognition accuracy for happy expressions of humans (Uono et al., 2011) and Nikola, respectively]. We speculate that this discrepancy was mainly attributable to low facial expression intensity for Nikola. Overall, the results indicate that Nikola can accurately exhibit emotional facial expressions of six basic emotions using a combination of AUs (Friesen and Ekman, 1983), although expression intensity is weak relative to human expressions.

## Study 3

In Study 3, we systematically changed the speed of Nikola’s dynamic facial expressions and asked naïve participants to evaluate the naturalness of the expressions’ speed, as in earlier psychological studies that used the dynamic stimuli of human facial expressions (Sato and Yoshikawa, 2004; Sato et al., 2013). Earlier studies that used human stimuli consistently reported that facial expressions that changed too slowly were generally rated as unnatural. Additionally, the effects of changing speeds differed across emotions, such that fast changes could be perceived as relatively natural for surprised expressions while slow changes were perceived as natural for sad expressions. We expected similar emotion-general and emotion-specific patterns for Nikola’s dynamic facial expressions.

### Materials and Methods

#### Participants

Thirty adult Japanese participants took part in this study (19 females; mean ± *SD* age, 37.0 ± 7.4 years). As in Study 2, the sample size was determined based on an *a priori* power analysis using G*Power software ver. 3.1.9.2 (Faul et al., 2007). Assuming an α level of 0.05, a power of 0.80, and a medium effect size (*f* = 0.25), the results indicated that 24 participants were required for the planned trend analyses (four levels). Participants were recruited through web advertisements distributed *via* CrowdWorks (Tokyo, Japan). After the procedures had been explained, all participants provided written informed consent to participate in the study, which was approved by the Ethics Committee of RIKEN. The experiment was performed in accordance with the Declaration of Helsinki.

#### Stimuli

**FIGURE 4:**
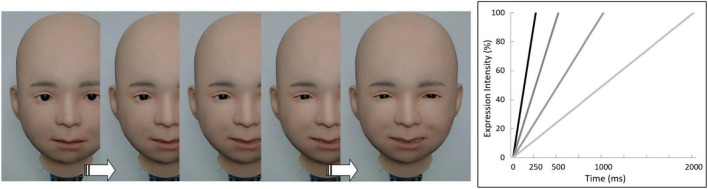
Illustration of the dynamic facial expression stimuli used in Study 3. **(Left)** Nikola’s face changed from a neutral expression to one of six emotional expressions. **(Right)** Schematic illustration of the four speed conditions.

A total of 24 videotapes of dynamic facial expressions produced by Nikola, depicting six basic emotions (anger, disgust, fear, happiness, sadness, and surprise), from onset (neutral face) to action apex (full emotional expression) at four speeds (total durations of 250, 500, 1,000, and 2,000 ms) were used as stimuli (Figure 4). The four speed conditions used in previous studies (Sato and Yoshikawa, 2004; Sato et al., 2013) were also employed herein to allow comparison of the findings between humans and androids. The utility of these speeds was also supported by our preliminary encoding study (some data were reported in Sato et al., 2019b), in which we videotaped emotional facial expressions produced in response to various scenarios and found that most expressions were produced within 250–2,000 ms. Similar data (production durations of 220–1,540 ms) were reported by a different group (Fiorentini et al., 2012). A decoding study reported that the presentation of dynamic facial expressions for 180, 780, and 3,030 ms produced divergent free-response recognition of facial expressions (Kamachi et al., 2001). As in Study 2, the AUs of emotional facial expressions were determined according to EMFACS (Friesen and Ekman, 1983). All AUs were controlled simultaneously. The facial expressions were video-recorded using a digital web camera (HD1080P; Logitech, Tokyo, Japan). The Supplementary Material provides video clips of these dynamic facial expression stimuli.

#### Procedure

As in Study 2, the experiment was conducted *via* the online Qualtrics platform (Seattle, WA, United States). The naturalness of dynamic changes in emotional facial expressions was rated, as in an earlier study (Sato and Yoshikawa, 2004). In each trial, four video clips of Nikola’s facial expressions of one of six basic emotions, at different speeds, were presented on the monitor one by one. The speed conditions were presented in randomized order, and the interval between each clip was 1,500 ms. Participants were provided with the target emotion label and instructed to evaluate each clip in terms of the naturalness of the speed with which the particular emotion changed, using a 7-point scale ranging from 1 (not at all natural) to 7 (very natural). No time limits were set, and participants were allowed to view the sequence repeatedly (by clicking a button) until they were satisfied with their ratings. Each emotion condition was presented twice in pseudo-randomized order, resulting in a total of 12 trials for each participant. Prior to the experiment, participants performed two practice trials.

#### Data Analysis

As in Study 2, the data were analyzed using JASP 0.14.1 software (JASP Team, 2020). The naturalness ratings were analyzed by repeated-measures ANOVA, with emotion (anger, disgust, fear, happiness, sadness, and surprise) and speed (total duration of 250, 500, 1,000, and 2,000 ms) as within-subjects factors. Because the assumption of sphericity was not met (Mauchly’s sphericity test, *p* < 0.05), the Huynh–Feldt correction was applied. Follow-up trend analyses were conducted on the effect of speed, to derive profiles of the changes in ratings across speed conditions. All results were considered statistically significant at *p* < 0.05.

### Results

**FIGURE 5:**
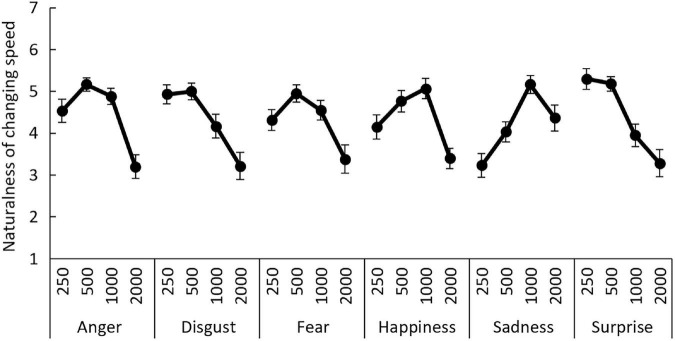
Mean (±*SE*) naturalness ratings for facial expressions of six emotions under the four speed conditions in Study 3.

The ANOVA for the naturalness ratings (Figure 5), with emotion and speed as within-subjects factors, revealed a significant main effect of speed, *F*_(1.52, 44.14)_ = 12.62, *p* = 0.000, and η^2^_*p*_ = 0.30. The interaction between emotion and speed was also significant, *F*_(7.42, 215.30)_ = 9.45, *p* = 0.000, and η^2^_*p*_ = 0.25. The main effect of emotion was not significant, *F*_(3.05, 88.40)_ = 0.84, *p* = 0.476, and η^2^_*p*_ = 0.03. Follow-up trend analyses of the main effect of speed indicated significant negative linear (i.e., faster changes were more natural) and quadratic (i.e., intermediate changes were the most natural) trends as a function of speed, *t*(87) = 3.98 and 4.68, respectively, *p*s = 0.000.

For the significant interaction, simple trend analyses of the speed effect were conducted for each emotion. For anger, disgust, and fear, the linear and quadratic negative trends as a function of speed were significant, *t*(87) = 4.21, 5.09, 5.29, 2.01, 2.78, and 3.52; *p* = 0.000, 0.000, 0.000, 0.048, 0.006, and 0.000, for anger-linear, anger-quadratic, disgust-linear, disgust-quadratic, fear-linear, and fear-quadratic, respectively. Only the negative quadratic trend was significant for happiness, *t*(87) = 4.94, *p* = 0.000. For sadness, the positive linear (i.e., slower changes were more natural) and negative quadratic trends were significant, *t*(87) = 3.72 and 2.94, *p* = 0.000 and 0.004, respectively. For surprise, only the negative linear trend reached significance, *t*(87) = 6.67, *p* = 0.000.

### Discussion

The results indicated that the naturalness ratings for dynamic changes in Nikola’s emotional facial expressions generally decreased with reduced speed of change. The results also revealed differences across emotions; for example, the ratings linearly decreased and increased depending on speed for surprised and sad expressions, respectively. These results are consistent with those of earlier studies that used dynamic human facial expressions (Sato and Yoshikawa, 2004; Sato et al., 2013). The results are also in line with studies showing that an android exhibiting dynamic facial expressions with the same temporal patterns as human facial expressions was rated as more natural than an android that did not exhibit such expressions (Ishi et al., 2017, 2019). Our results demonstrate that the temporal aspects of Nikola’s facial expressions can transmit emotional messages, similar to those of humans.

## General Discussion

In summary, the results of Study 1 confirmed that Nikola can produce AUs associated with prototypical facial expressions. Study 2 verified that Nikola can exhibit facial expressions of six basic emotions that can be accurately recognized by naïve participants. The results of Study 3 revealed that Nikola can exhibit dynamic facial expressions with temporal patterns that transmit emotional messages, as in human facial expressions. Collectively, these results support the validity of the spatial and temporal characteristics of the emotional facial expressions of our new android.

These results have practical implications. First, in terms of basic research, androids like Nikola represent important tools for psychological experiments examining face-to-face emotional interactions with high ecological validity and control. Several methods have been employed to conduct such experiments, each of which has specific advantages and disadvantages. Most studies in the literature have presented pre-recorded photographs or videos of others’ emotional expressions (e.g., Dimberg, 1982). Although this method provides a high level of control, its ecological validity is not particularly high (for a review, see Shamay-Tsoory and Mendelsohn, 2019); a recent study indicated that subjective and physiological responses to pre-recoded videos of facial expressions differed from those to live facial expressions (Hsu et al., 2020). Live emotional interactions between two participants are ecologically valid (e.g., Bruder et al., 2012; Riehle et al., 2017; Golland et al., 2019); however, such interactions are difficult to control, and the correlational nature of this approach makes it difficult to establish causality in terms of psychological mechanisms. Confederates are commonly used in social psychology (e.g., Vaughan and Lanzetta, 1980); although this approach has high ecological validity, serious disadvantages include difficulty in controlling confederates’ non-verbal behaviors (for reviews, see Bavelas and Healing, 2013; Kuhlen and Brennan, 2013). Interactions with virtual agents may promote both ecological validity and control (Parsons, 2015; Pan and Hamilton, 2018); however, virtual agents are obviously not physically present, which may limit ecological validity to some degree. Several studies have reported that physically present robots elicited greater emotional responses than virtual agents (e.g., Bartneck, 2003; Fasola and Mataric, 2013; Li et al., 2019; for a review, see Li, 2015). Taken together, our data suggest that androids like Nikola, which are human-like in appearance and facial expressions, and can physically coexist with humans, are valuable research tools for ecologically valid and controlled research on facial emotional interaction. Moreover, like several other advanced androids (e.g., Glas et al., 2016; Ishi et al., 2017, 2019), Nikola has the ability to talk with prosody, which can facilitate multimodal emotional interactions (Paulmann and Pell, 2011). Androids can also utilize advanced artificial intelligence (for reviews, see Krumhuber et al., 2021; Namba et al., 2021) to sense and analyze human facial expressions. We expect that androids will be a valuable tool in future psychological research on human emotional interaction.

Second, regarding future applications to real-life situations, our results suggest that androids like Nikola have the potential to transmit emotional messages to humans, and in turn promote human wellbeing. Android interactions may be useful in a wide range of situations, including elder care, behavioral interventions, counseling, nursing, education, information desks, customer service, and entertainment. For example, an earlier study has reported that a humanoid robot, which was controlled by manipulators and exhibited facial expressions of various emotions, was effective in comforting lonely older people (Hoorn et al., 2016). The researchers found that the robot satisfied users’ needs for emotional bonding as a social entity, while retaining a sense of privacy as a machine (Hoorn et al., 2016). With regard to behavioral interventions, several studies showed that children with autism spectrum disorder preferred robots and androids to human therapists (e.g., Adams and Robinson, 2011; for a review, see Scassellati, 2007). We expect that increasing their ability for emotional interactions would enhance androids’ value in future real-life applications.

Our results also have theoretical implications. Our findings could be regarded as constructive support for psychological theories that certain configurations of AUs can indicate emotional facial expressions (Ekman and Friesen, 1975) and that temporal patterns of facial expressions might transmit emotional information (Sato and Yoshikawa, 2004). Other ideas regarding human emotional interactions may also be verifiable through android experiments. The construction of effective android software and hardware requires that the mechanisms of psychological theories be elucidated. We expect that this constructivist approach to developing and testing androids (Ishiguro and Nishio, 2007; Minato et al., 2007) will be a useful methodology for understanding the psychological mechanisms underlying human emotional interaction.

Some limitations of this study should be acknowledged. First, as described above, the number and intensity of Nikola’s AUs is not comparable with those of humans owing to technical limitations related to the number of actuators and skin materials. Specifically, because silicone skin does not possess elastic qualities comparable with human skin (Cabibihan et al., 2009), creating natural wrinkles in Nikola’s face is difficult. Previous psychological studies have shown that nose wrinkling (i.e., AU 9) was associated with the recognition of disgust (Galati et al., 1997), while eye corner wrinkles (i.e., AU 6) improved the recognition of happy and sad expressions (Malek et al., 2019), suggesting the importance of wrinkles in emotional expressions. Future technical improvements will be required to realize richer and stronger emotional facial expressions.

Second, we used only controlled and explicit measures of the recognition of emotional facial expressions, including emotion labeling and naturalness ratings of speed changes; we did not measure automatic and/or reactive responses to facial expressions. Several previous studies have shown that emotional facial expressions induced stronger subjective (e.g., emotional arousal; Sato and Yoshikawa, 2007a) and physiological (e.g., activation of the sympathetic nervous system; Merckelbach et al., 1989) emotional reactions compared with non-facial stimuli. Other studies reported that observing emotional facial expressions automatically induced facial mimicry (e.g., Dimberg, 1982). Because Nikola’s eyeballs contain video cameras, it may be possible to videorecord participants’ faces to reveal externally observable facial mimicry, which cannot be accomplished in human confederates without specialized devices (Sato and Yoshikawa, 2007b). Investigation of these automatic and reactive measures represents a key avenue for future research.

Third, we only tested the temporal patterns of Nikola’s facial expressions in Study 3, by manipulating speed at four levels; thus, the optimal temporal characteristics of Nikola’s dynamic facial expressions remain to be identified. A previous psychophysical study has investigated this issue using generative approaches (Jack et al., 2014). The researchers presented participants with a large number of dynamic facial expressions of virtual agents with randomly selected AU sets and temporal parameters (e.g., acceleration) and asked them to identify the emotions being displayed. Mathematical modeling revealed the optimal spatial and temporal characteristics of facial expressions of various emotions. Research using similar data-driven approaches could reveal more fine-grained temporal, as well as spatial, characteristics of the dynamic facial expressions of Nikola.

Finally, although we constructed Nikola’s facial expressions according to basic emotion theory (Ekman and Friesen, 1975), the relationships between facial expressions and psychological states can be investigated from various perspectives. For example, Russell (1995, 1997) has proposed that facial expressions are associated not with basic emotions, but rather with core affective dimensions of valence and arousal. Fridlund and his colleagues proposed that facial expressions indicate not emotional states, but rather social messages (Fridlund, 1991; Crivelli and Fridlund, 2018). Investigation of these perspectives on facial expressions using androids is a key topic for future research.

## Data Availability Statement

The original contributions presented in the study are included in the article/Supplementary Material, further inquiries can be directed to the corresponding author.

## Ethics Statement

The studies involving human participants were reviewed and approved by the RIKEN. The patients/participants provided their written informed consent to participate in this study.

## Author Contributions

WS and TM designed the research. WS, SNa, DY, SNi, and TM obtained the data. WS and SNa analyzed the data. WS, SNa, DY, SNi, CI, and TM wrote the manuscript. All authors contributed to the article and approved the submitted version.

## Conflict of Interest

SNi was employed by the company Nippon Telegraph and Telephone Corporation. The remaining authors declare that the research was conducted in the absence of any commercial or financial relationships that could be construed as a potential conflict of interest.

## Publisher’s Note

All claims expressed in this article are solely those of the authors and do not necessarily represent those of their affiliated organizations, or those of the publisher, the editors and the reviewers. Any product that may be evaluated in this article, or claim that may be made by its manufacturer, is not guaranteed or endorsed by the publisher.

## Supplementary Material

Supplementary Figure 1 Video clips of facial action units (AUs).

Supplementary Figure 2 Video clips of the dynamic facial expression stimuli used in Study 3.

Supplementary Data 1 Datasheet for Studies 2 and 3.
